# The intra-articular administration of triamcinolone hexacetonide in the treatment of osteoarthritis. Its effects in a naturally occurring canine osteoarthritis model

**DOI:** 10.1371/journal.pone.0245553

**Published:** 2021-01-20

**Authors:** João C. Alves, Ana Santos, Patrícia Jorge, Catarina Lavrador, L. Miguel Carreira

**Affiliations:** 1 Divisão de Medicina Veterinária, Guarda Nacional Republicana (GNR), Lisbon, Portugal; 2 MED – Mediterranean Institute for Agriculture, Environment and Development, Instituto de Investigação e Formação Avançada, Universidade de Évora, Pólo da Mitra, Évora, Portugal; 3 Faculty of Veterinary Medicine, University of Lisbon (FMV/ULisboa), Lisbon, Portugal; 4 Interdisciplinary Centre for Research in Animal Health (CIISA) – University of Lisbon, (FMV/ULisboa), Lisbon, Portugal; 5 Anjos of Assis Veterinary Medicine Centre (CMVAA), Barreiro, Portugal; University of Life Sciences in Lublin, POLAND

## Abstract

**Objective:**

To evaluate the effect of an intra-articular (IA) administration of triamcinolone hexacetonide, compared with saline.

**Patients and methods:**

Forty (N = 40) hip joints were randomly assigned to a treatment group (THG, n = 20, receiving IA triamcinolone hexacetonide) and a control group (CG, n = 20, receiving IA saline). On treatment day (T0), and at 8, 15, 30, 90 and 180 days post-treatment, weight distribution, joint range of motion, thigh girth, digital thermography, radiographic signs, synovial fluid interleukin-1 and C-reactive protein levels were evaluated. Data from four Clinical Metrology Instruments was also gathered. Results were compared Repeated Measures ANOVA, with a Huynh-Feldt correction, Paired Samples T-Test or Wilcoxon Signed Ranks Test. A Kaplan-Meier test was performed to compare both groups, with p<0.05.

**Results:**

Joints were graded as mild (65%), moderate (20%) and severe (15%). Patients of both sexes, with a mean age of 6.5±2.4 years and bodyweight of 26.7±5.2kg, were included. No differences were found between groups at T0. Comparing THG to CG, weight distribution showed significant improvements in THG from 8 (p = 0.05) up to 90 days (p = 0.01). THG showed lower values during thermographic evaluation in the Lt view (p<0.01). Pain and function scores also improved from 30 to 180 days. Increasing body weight, age, and presence of caudolateral curvilinear osteophyte corresponded to worse response to treatment. Results of the Kaplan Meier test showed significant differences between groups, with THG performing better considering several evaluations and scores.

**Conclusion:**

THG recorded significant improvements in weight-bearing and in with the considered CMIs, particularly pain scores. Lower thermographic values were registered in THG up to the last evaluation day. Age, sex, and radiographic findings did significantly influenced response to treatment.

## Introduction

Osteoarthritis (OA) is a disease transversal to all mammals and a source of chronic pain. For that reason, it represents a considerable burden to societies, representing a large investment in healthcare, while reducing productivity and quality of life [1–3]. Since OA is symptomatic only in the affected joint while, at the same time, lacking obvious extra-articular manifestations, it is well suited to administer local therapy by intra-articular (IA) injection [4, 5]. The changes that occur in slowly progressive spontaneous dog OA closely match those of human OA, while maintaining the same life stages that go by at a faster progression rate, and sharing many of the same environmental conditions. For those reasons, the naturally occurring canine model is considered the closest to a gold standard [6–11].

The medical approach to OA aims at slowing disease progression while relieving symptoms, particularly pain [9, 12]. IA corticosteroids have been used for several decades to palliate pain and inflammation associated with OA and of joint’s surrounding tissues [13, 14]. Its use should be especially considered in patients with moderate to severe pain, nonresponding to oral analgesic/non-steroidal anti-inflammatory drugs. A human systematic review has deemed triamcinolone more effective than betamethasone and methylprednisolone [15]. Triamcinolone hexacetonide (TH), in particular, is described as able to provide pain relief and improved mobility for prolonged periods [16, 17]. In a canine model of OA, animals treated with IA TH showed a significant reduction of osteophyte size compared with a control group. At the histological level, TH significantly reduced the severity of OA structural changes of cartilage and had no deleterious effects on normal cartilage [18]. By effect is obtained through a dose-dependent reduction in the cartilage proteolytic enzyme stromelysin, interleukin 1β, and the oncogenes c-fos and c-myc, which are involved in the metalloproteinases synthesis [19]. In human patients, the mean duration of effect of TH in patients with OA is around seven months [20].

Since pain and functional limitations are the most relevant clinical signs of OA, clinical trials and studies need to assess these parameters in order to evaluate patients and assessment of response to treatment [21–23]. Clinical metrology instruments (CMI) represent a patient-centred approach, and the most commonly used are the Canine Brief Pain Inventory (CBPI, divided in a pain severity score—PSS, and a pain interference score—PIS) and the Liverpool Osteoarthritis in Dogs (LOAD). Further validated CMIs include the Hudson Visual Analogue Scale (HVAS), and the Canine Orthopaedic Index (COI, divided into four scores: stiffness, gait, function and quality of life—QOL). As a whole, CMIs complement the evaluation of the multi-dimensional experience that is OA related pain [9, 23–31]. Typically, OA pain is localized and related to movement or weight-bearing of the affected joints, and affected patients commonly bear less weight on a painful limb. Evaluating weight distribution through stance analysis is a sensitive evaluation canine lameness [22, 23, 32]. Additional functional evaluations aim at assessing activity levels and mobility impairments [26]. Pedometry is a simple and inexpensive method to assess mobility levels, which can measure ambulatory activity with an acceptable level of accuracy [33]. Additional clinical measurements include the examination of muscle masses and evaluation of the joint range of motion, which are consistently reduced and restricted in OA patients [34–37].

Imaging plays a key role in the assessment of patients with joint disease and, in cases of hip OA, the ventrodorsal (VD) hip extended view is the most common pelvic radiographic projection. The ventrodorsal flexed view, also called frog-legged view (FL), useful for further evaluation of the presence of the circumferential femoral head osteophyte (CFHO) and caudolateral curvilinear osteophyte (CCO), radiographic findings related with the development of clinical signs [38–43]. By correlating changes in temperature patterns with various disease, degenerative or injury processes, digital thermography can provide a reproducible diagnostic tool [44–46]. This diagnosis modality can differentiate normal from osteoarthritis subjects [47, 48].

Since OA is a low-grade inflammatory disease, the analysis of synovial fluid (SF) can add additional information regarding the disease’s characterization [1]. Interleukin 1 (IL-1) is the most important proinflammatory catabolic cytokine in OA, with a highly potent capability of inducting cartilage degradation and relation with lameness duration [49–51]. C-reactive protein (CRP) can be produced at the level of the inflamed tissues, and its shifts occur from a very early stage [52, 53]. It has been highly associated with knee OA in humans [54].

The goal of this study is to compare the effect of triamcinolone hexacetonide to a control group in the management of OA in a naturally occurring canine model, using several outcome assessment modalities. We hypothesize the intra-articular administration of triamcinolone hexacetonide will be able to reduce the clinical signs of OA, compared to a control group.

## Materials and methods

The study protocol was approved by the ethical review committee of the Universidade de Évora (ORBEA, approval n° GD/32055/2018/P1, September 25^th^, 2018), and complies with the ARRIVE guidelines. Written, informed consent was obtained from the Institution responsible for the animals. Twenty dogs were selected based on medical history records, physical, orthopaedic, neurological and radiographic examinations compatible with hip OA, and the sample comprised forty (N = 40) joints of twenty active police working dogs with bilateral hip OA. To be included in the study, patients should be over two years, have a bodyweight over 20kg and should not have received any medication or nutritional supplement for over six weeks before enrolment in the study. Patients with any other documented or suspected orthopaedic or neurological disease, or additional concomitant disease (ruled out through physical examination, complete blood count, and serum chemistry profile), were excluded.

In a double-blinded study, dogs with affect joints were randomly assigned to a control group (CG, n = 20 joints) or a treatment group (THG, n = 20 joints). Evaluations were conducted on days 0 (treatment day), 8, 15, 30, 90 and 180. Days were counted from treatment day (day 0). An outline of all procedures on each evaluation day is presented in Table 1. The same researcher performed all evaluations.

**Table 1:** Procedures conducted in each day. Days are counted from treatment day.

Procedure	Day					
	0	8	15	30	90	180
Treatment	X					
Digital Thermography	X	X	X	X	X	X
Digital radiography	X			X	X	X
Stance analysis	X	X	X	X	X	X
Pedometer	X	X	X	X	X	X
Goniometry	X	X	X	X	X	X
Thigh girth measurement	X	X	X	X	X	X
Clinical Metrology Instruments	X	X	X	X	X	X
Synovial fluid CRP	X	X		X	X	X
Synovial fluid IL-1	X	X		X	X	X

CMI—Clinical Metrology Instruments; CRP—C-Reactive Protein; IL-1 —Interleukin 1.

On treatment day, patients in CG received an IA administration of 2ml of 0.9%NaCl. On the same day, patients in THG received an IA administration of 20mg in a volume of 1 ml of triamcinolone hexacetonide (Bluxam, Riemser Pharma). IA administrations and radiographic examination were conducted under light sedation, induced with a combination of medetomidine (0.01mg/kg) and buthorphanol (0.1mg/kg), given intravenously. After all procedures were conducted, sedation was reversed with atipamezole (100–150μg/kg), administered intramuscularly. Both a VD extended legs and FL views were obtained. On the VD view, the presence of the following findings was assessed; an irregular wear on the femoral head, making it misshapen and with a loss of its rounded appearance; a flattened or shallow acetabulum, with irregular outline; CCO; new bone formation on the acetabulum and femoral head and neck; a worn away angle formed at the cranial effective acetabular rim; subchondral bone sclerosis along the cranial acetabular edge; and CFHO [43, 55, 56]. For the IA administration, patients were positioned in lateral recumbency, with the joint of interest uppermost. A window of 4x4cm in the area surrounding the greater trochanter was clipped and aseptically prepared. The limb was then placed in a neutral, parallel to the table position and a 21-gauge with 2.5” length needle was introduced just dorsal to the greater trochanter, perpendicular to the long axis of the limb until the joint was reached [57]. Confirmation of correct needle placement was obtained through the collection of SF. As much SF as possible was aspirated and kept for the posterior determination of IL-1β and CRP concentrations, and the treatment or saline was administered.

Evaluation of weight distribution was carried out with a weight distribution platform (Companion Stance Analyzer; LiteCure LLC^®^, Newark, Delaware, United States), following manufacturer’s guidelines. The equipment was placed in the centre of a room, at least 1 meter from the walls. It was also calibrated at the beginning of each day and zeroed before each data collection. For the evaluation, patients were led to stand on the platform, with one foot on each quadrant, with their heads facing forward. The left-right symmetry index was calculated with the following formula: symmetry index = [(WB_R_-WB_L_)/((WB_R_+WB_L_)x0.5)]x100 [28, 58], where WB_R_ is the value of weight-bearing for the right pelvic limb, and WB_L_ is the value of weight-bearing for the left pelvic limb. Negative values were made positive. Additionally, we considered a deviation from the normal 20% weight-bearing for a pelvic limb [59], calculated by subtracting WB to 20.

Pedometers were worn around the patient’s neck, attached to an adjustable lightweight collar, to measure ambulatory activity and mobility levels [60]. They were worn for a week before the first evaluation time, to order to establish a baseline value, and before each evaluation time. Mean daily counts were considered, calculated by dividing the register number of steps by the number of considered days. In a quiet room, with as much time as needed to answer all items, trainers completed a copy of HVAS, CBPI, COI and LOAD, in sequence by the same handler at all evaluation days.

**Fig 1:**
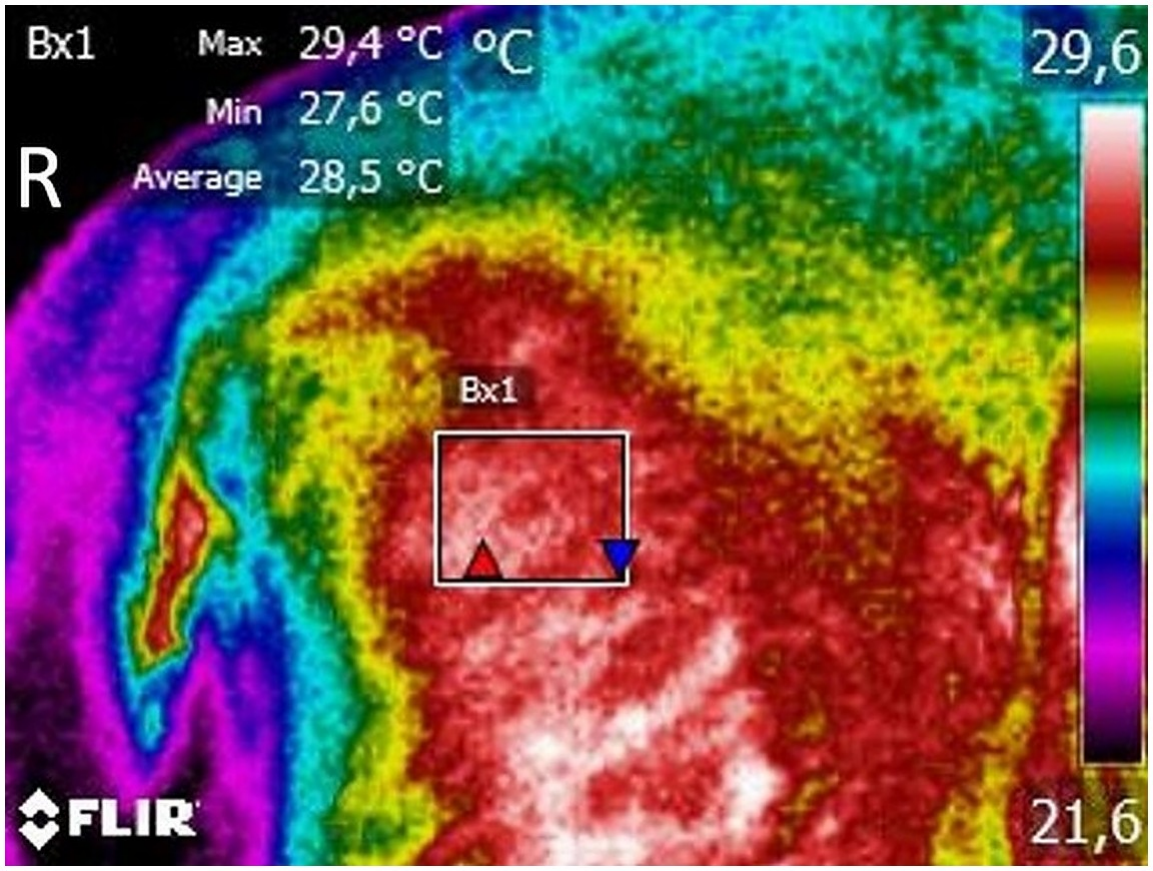
A lateral view of a dog with moderate osteoarthritis, with the greater trochanter in the centre, at a distance of 60 cm. The range of temperature was set at 15–40°C and emissivity at 0.98. Thermographic images were analyzed with a Rainbow HC colour pallet. A temperature box is placed on the anatomical area of the hip joint.

For the digital thermography evaluation, animals were kept for 30 minutes in a room with controlled temperature, at 21°C. During this period, they were allowed to walk around the room calmly. A dorsoventral image was obtained with patients in a symmetrical upright standing, including the area from the last lumbar to the first coccygeal vertebrae, at a distance of 60 cm [61]. A lateral view was also obtained, with the greater trochanter in the centre, at the same distance. All images were taken with a FLIR ThermaCAM E25^®^ model (FLIR Systems, Wilsonville, Oregon, United States). The posterior analysis was conducted with free software (Tools, FLIR Systems, Inc), using a rainbow colour pallet. Temperature boxes were placed on the anatomical area of the hip joint, to determine mean and maximal temperatures (Fig 1).

Both thigh girth and joint range of motion were determined with the patient in lateral recumbency. A Gullick II measuring tape was used to evaluate thigh girth, at a distance of 70% thigh length, measured from the tip of the greater trochanter, with an extended leg [62]. Hip joint ROM was then determined with a goniometer at extension and flexion with a flexed stifle [63].

Determination of IL-1β and CRP concentrations were made using Fuji Dri-Chem Slides VC-CRP PS (FUJIFILM Europe GmbH), read with a DRIChem NX500i (FUJIFILM Europe GmbH), and a DuoSet Ancillary Canine IL-1β Reagent kit (R&D Systems, United Kingdom), read with a FLUOstar OPTIMA (BMG Labtech).

After treatment, animals were rested for three consecutive days and resumed their regular activity over five days. On days 1 and 3 after the procedure, the veterinarian examined all patients in order to determine existing signs of exacerbated pain, persistent stiffness of gait and changes in posture. If no complaints were registered, the animal could resume its normal activity [64, 65].

Normality was assessed with a Shapiro-Wilk test. Different group’s results were compared in each evaluation day, and each measured parameter was compared with the result observed at treatment day. To assess the effect of different parameters on the patients’ clinical evolution, results were compared by sex, age and different cut off values for body weight with Repeated Measures ANOVA, with a Huynh-Feldt correction, Paired Samples T-Test, or Wilcoxon Signed Ranks Test. A Kaplan-Meier test was performed to evaluate the time to return to baseline values of symmetry index and CMI scores, compared with the Breslow test. All results were analyzed with IBM SPSS Statistics version 20, and a significance level of p<0.05 was set.

## Results

The sample included 40 hip joints (n = 20 left and n = 20 right) of active police working dogs with a mean age of 6.5±2.4 years and bodyweight of 26.7±5.2kg. Both sexes (male n = 28, female n = 12) and four breeds were represented: German Shepherd Dogs (n = 8), Belgian Malinois Shepherd Dogs (n = 6), and Dutch Shepherd Dog (n = 6). At T0, 26 joints were classified as mild (65%), 8 as moderate (20%) and 6 as severe (15%), according to the Orthopedic Foundation for Animals hip grading scheme. No differences were found between groups at the initial evaluation. After the initial rest period, all animals resumed normal activity, with similar workload and movement compared to that before treatment.

### Clinical and laboratorial findings

**Fig 2:**
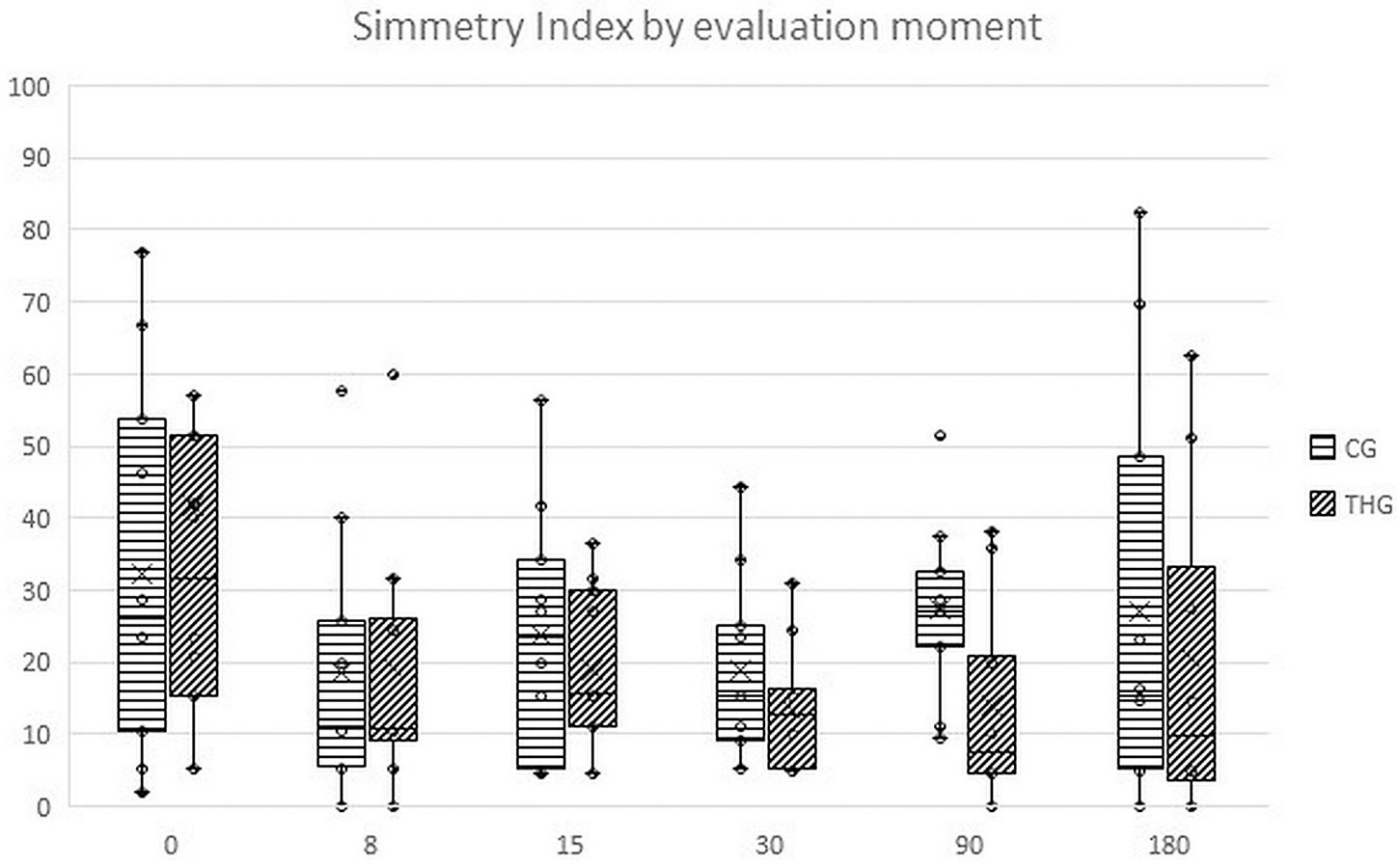
Overall evolution of symmetry index in the control group (CG) and treatment group (THG). Box plots represent the median, 25th and 75th percentiles, and whiskers represent 10th and 90th percentiles.

**Fig 3:**
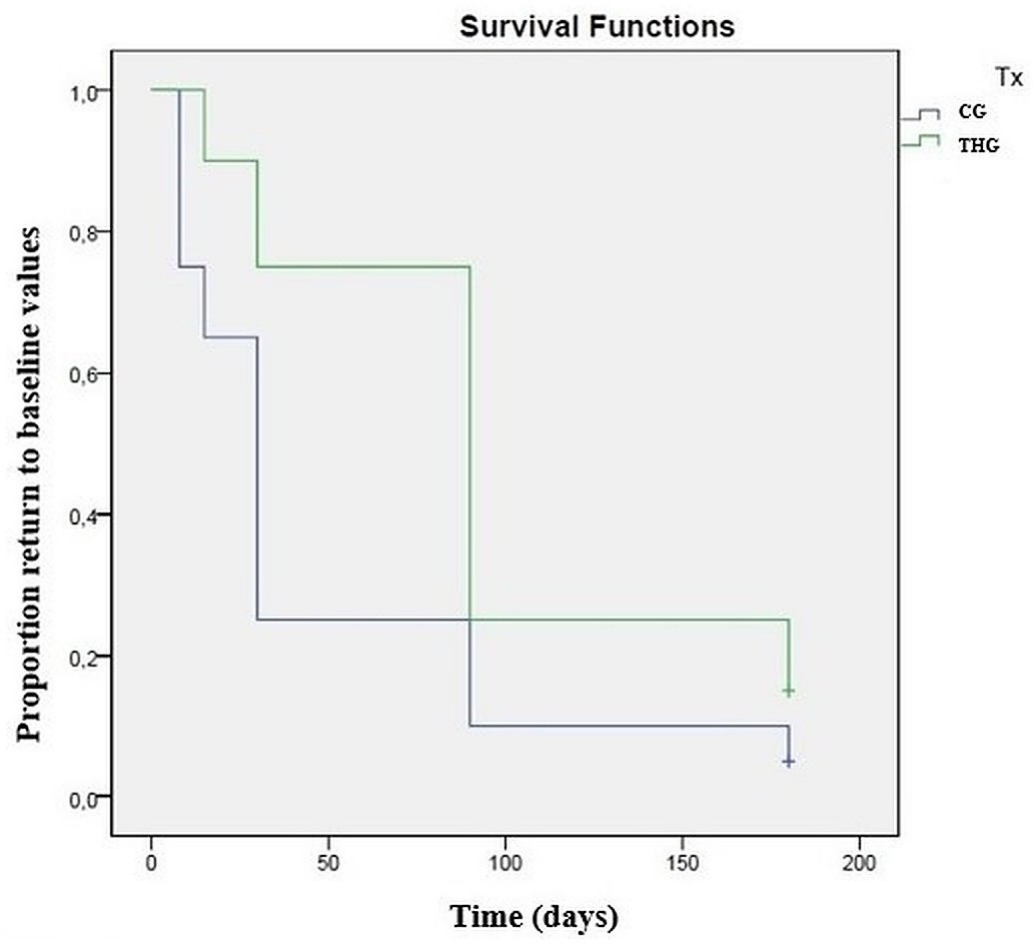
Kaplan-Meier curve demonstrating a significant difference between the control group (CG) and triamcinolone hexacetonide group (THG) in time for the symmetry index to return to baseline values (p = 0.003).

**Fig 4:**
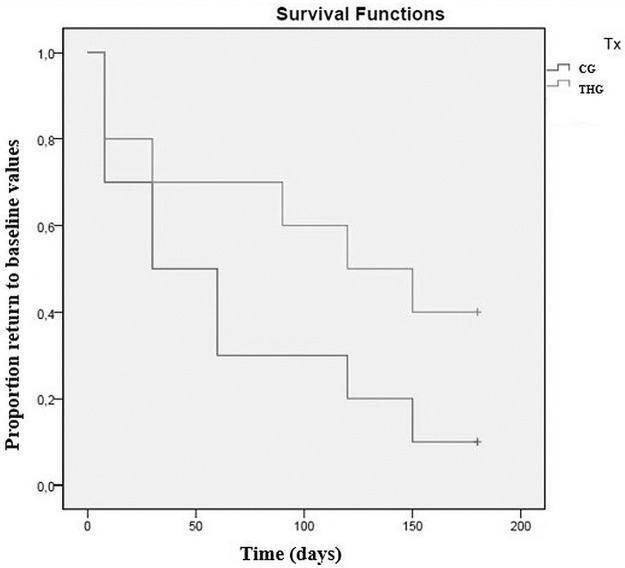
Kaplan-Meier curve demonstrating a significant difference between the control group (CG) and triamcinolone hexacetonide group (THG) in time for function score to return to baseline values (p = 0.046*).

**Fig 5:**
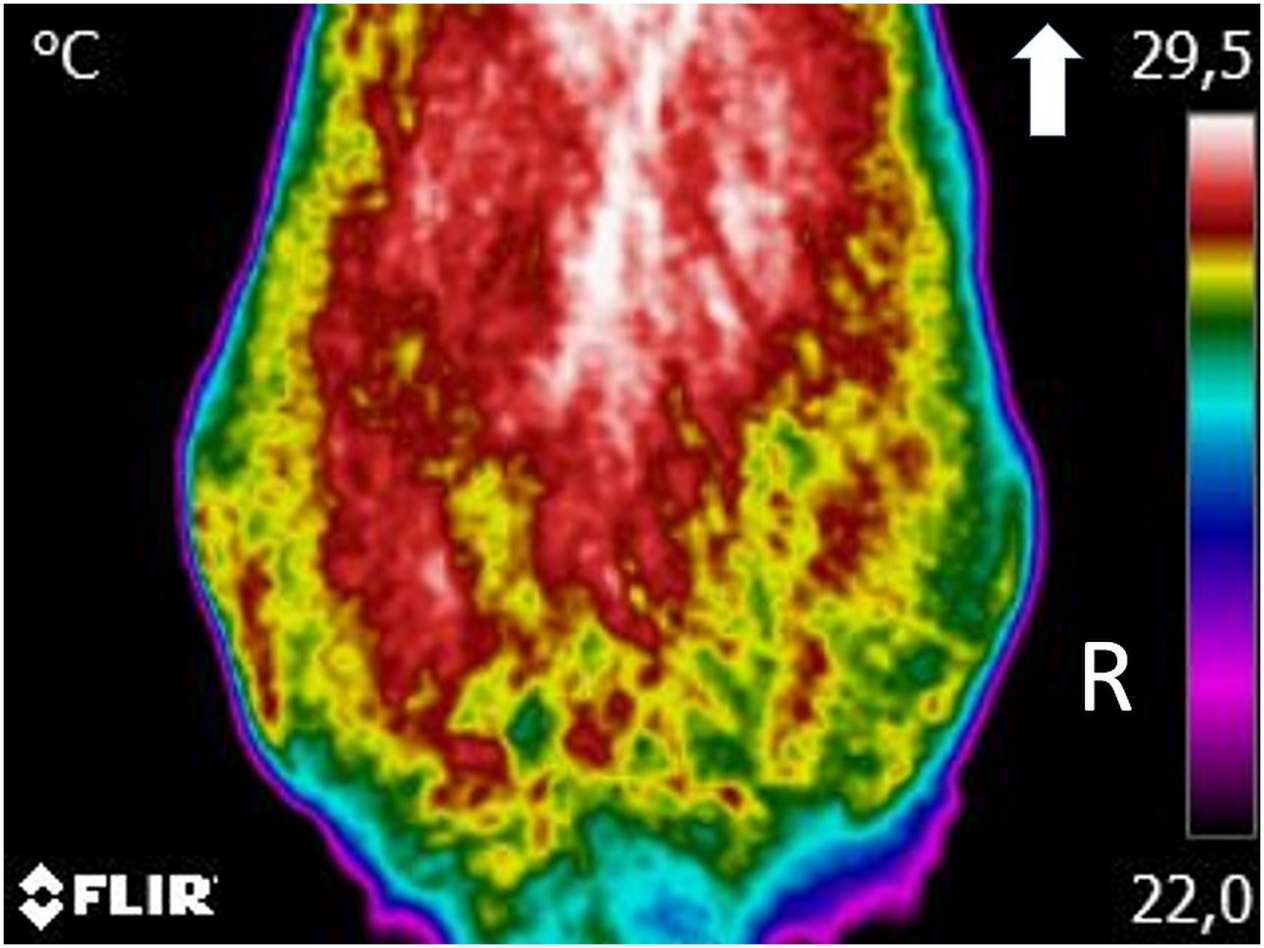
A dorsoventral view of a dog with moderate osteoarthritis, including the area from the last lumbar vertebra to the first coccygeal vertebra at a minimum, at a distance of 60 cm. Arrow indicates cranial direction. The range of temperature was set at 15–40°C and emissivity at 0.98. Thermographic images were analyzed with a Rainbow HC colour pallet.

Values recorded for different assessments at the initial evaluation, and its variations throughout the study, for THG and CG, are presented in Table 2. Comparing results between groups with repeated measures ANOVA with a Huynh-Feldt correction, significant differences between groups were found concerning body weight (F(2.8,140) = 4.2, p<0.01), deviation (F(4.8,109) = 2.8, p = 0.02), symmetry index (F(2.8,77.8) = 7.5, p<0.01), mean temperature on a DV view (F(3.9,93.9) = 6.6, p<0.01), maximal temperature on a DV view (F(3.6,86.2) = 6.9, p<0.01), mean temperature on a Lt view (F(4.5,113.2) = 26.7, p<0.01), maximal temperature on a Lt view (F(4.1,101.6) = 96.2, p<0.01), joint flexion (F(4.9,146.7) = 19.5, p<0.01), IL-1 synovial concentration (F(1.9,58.3) = 4.9, p = 0.02). Significant differences were observed between groups regarding CMI scores, specifically PSS (F(5,120) = 2.4, p<0.05), PIS (F(5,120) = 2.6, p = 0.03) and function (F(2.8,69.2) = 2.4, p = 0.04). Evolution of the symmetry index in CG and THG is presented in Fig 2. Results of the Kaplan Meier test are presented in Table 3. Kaplan Meier curves for symmetry index and function score are presented in Figs 3 and 4, respectively. A dorsoventral digital thermography view is presented in Fig 5.

**Table 2:** Mean values (±standard deviation) of different parameters evaluated at the initial evaluation and throughout the study.

**Modality**		**Treatment day**				**8 days**							**15 days**									
		**CG**		**THG**		**CG**			**THG**			**P**	**CG**			**THG**			**P**			
		**mean**	**SD**	**mean**	**SD**	**mean**	**SD**	**p**	**mean**	**SD**	**p**		**mean**	**SD**	**p**	**mean**	**SD**	**p**				
Goniometry	Flexion (°. mean±SD)	55.0	4.4	57.0	4.1	55.3	3.7	<0.01*	55.3	4.7	0.09	1.0	57.2	5.2	0.14	58.0	5.3	0.53	1.0			
	Extension (°. mean±SD)	151.2	3.9	148.0	8.8	149.9	4.6	0.95	149.4	4.5	0.41		151.1	3.5	0.07	151.3	5.1	<0.05*	1.0			
	Thigh girth (cm. mean±SD)	31.2	2.6	30.4	3.4	31.1	3.3	0.94	30.8	3.1	1.0	0.49	31.1	2.9	0.86	32.6	3.5	<0.01*	0.58			
	Pedometer (daily steps±SD)	1445.7	755.7	1539.6	397.3	829.5	931.3	0.58	989.8	1554.6	0.85		606.0	309.5	0.15	1215.1	793.8	0.03*	1.0			
CMI	HVAS (0–10)	6.8	1.2	5.7	1.9	6.7	1.5	0.48	6.4	1.7	0.19	0.16	6.8	1.2	0.6	6.3	1.6	0.72	0.16			
	CBPI—PSS (0–10)	3.1	1.9	4.2	2.8	3.4	2.3	0.69	2.9	1.8	0.03*	<0.05*	3.7	2.8	0.2	3.2	1.9	0.04*	0.04*			
	CBPI—PIS (0–10)	3.2	2.2	4.8	3.3	3.4	2.1	0.01*	3.3	2.1	0.04*	0.02*	3.6	2.1	0.01*	3.5	2.1	0.03*	0.01*			
	COI—Stiffness (0–16)	3.4	3.4	6.8	4.2	4.1	3.3	0.31	5.5	3.6	0.38	0.51	4.1	3.2	0.31	5.1	4.0	0.46	10.19			
	COI—Function (0–16)	3.6	4.1	6.3	5.7	4.1	4.0	0.64	4.1	4.0	0.21	<0.05*	4.4	5.5	0.72	3.8	3.9	0.75	0.03*			
	COI—Gait (0–20)	4.7	5.2	10.5	5.9	5.4	6.1	0.29	7.3	4.7	0.18	0.21	5.8	4.3	0.02*	6.8	5.6	<0.05*	0.19			
	COI—QOL (0–12)	4.5	2.6	6.2	3.9	4.6	2.7	0.62	5.5	3.0	0.28	0.85	4.7	2.9	0.06	4.4	3.6	0.56	0.09			
	COI—Overall score (0–64)	16.4	14.7	29.8	19.1	18.2	13.8	0.22	23.8	14.6	0.19	0.48	18.6	13.8	0.14	21.0	16.7	0.83	0.19			
	LOAD (0–52)	13.6	10.5	23.2	14.1	14.4	12.7	1.0	18.3	12.0	0.98	0.33	14.3	10.7	0.83	18.0	12.3	0.88	0.14			
Digital Thermography	DV (°. mean±SD)	24.7	1.9	25.3	0.6	25.2	1.3	0.01*	25.3	0.6	0.08	<0.01*	24.4	1.6	0.61	23.4	2.5	0.03*	1.0			
	DV max (°. mean±SD)	26.3	1.9	26.4	1.6	25.8	1.0	0.06	24.9	1.6	0.24	<0.01*	26.7	1.6	0.97	24.6	2.5	0.03*	1.0			
	Lt (°. mean±SD)	28.7	2.7	26.5	1.9	31.6	2.1	<0.01*	30.5	2.8	<0.01*	<0.01*	29.7	2.9	<0.01*	28.5	4.0	0.02*	<0.01*			
	Lt max (°. mean±SD)	31.9	3.1	30.4	4.1	34.9	1.0	<0.01*	34.6	1.1	<0.01*	<0.01*	34.9	0.8	<0.01*	34.0	1.9	<0.01*	<0.01*			
Synovial fluid	IL-1 (pg/mL. mean±SD)	170.9	120.4	208.5	95.2	72.3	42.4	<0.01*	98.4	80.8	0.04*	0.04*	-	-	-	-	-	-	-			
	CRP (mg/mL. mean±SD)	0.4	1.0	2.5	3.5	0.3	1.2	<0.01*	0.0	0.0	0.18	1.0	-	-	-	-	-	-	-			
Weight-bearing	Symmetry Index (mean±SD)	24.7	20.3	53.9	50.4	18.7	17.1	0.06	19.2	18.1	<0.05*	<0.05*	23.9	16.3	0.18	18.9	10.9	0.01*	0.03*			
	Deviation (mean±SD)	2.8	3.6	4.7	4.4	2.78	1.987	0.3	2.1	1.9	0.08	0.43	2.94	2.127	0.47	2.2	1.6	<0.05*	0.02*			
**Modality**		**30 days**							**90 days**							**180 days**						
		**CG**			**THG**			**P**	**CG**			**THG**			**P**	**CG**			**THG**			**P**
		**mean**	**SD**	**P**	**mean**	**SD**	**P**		**mean**	**SD**	**p**	**mean**	**SD**	**p**		**mean**	**SD**	**P**	**mean**	**SD**	**P**	
Goniometry	Flexion (°. mean±SD)	53.6	2.9	0.11	51.8	3.9	<0.01*	<0.01*	52.7	2.9	0.02*	52.0	3.8	<0.01*	<0.01*	51.6	2.2	0.00*	49.3	4.3	<0.01*	<0.01*
	Extension (°. mean±SD)	150.8	3.4	0.06	152.3	3.7	0.09	1.0	150.8	2.9	0.07	151.9	3.0	<0.01*	0.23	151.3	2.9	0.17	150.2	3.7	0.26	1.0
	Thigh girth (cm. mean±SD)	30.6	2.7	0.39	29.5	3.1	0.25	0.44	31.6	2.7	0.54	31.5	3.7	0.09	0.43	31.5	2.2	0.2	30.2	3.7	0.96	0.32
	Pedometer (daily steps±SD)	594.5	663.4	0.48	747.9	548.2	0.65	0.15	451.9	463.0	0.4	410.8	497.4	0.15	0.08	434.9	455.8	0.2	376.0	263.3	<0.01*	0.63
CMI	HVAS (0–10)	6.4	1.4	0.14	6.3	1.9	0.64	0.17	6.6	1.7	0.22	6.5	1.3	0.84	0.21	6.5	1.4	0.04*	6.4	1.6	0.13	0.12
	CBPI—PSS (0–10)	3.7	2.6	0.03*	3.8	2.6	0.02*	0.01*	4.1	2.9	0.02*	3.2	2.1	0.57	0.32	3.6	3.1	0.02*	3.6	2.5	0.98	0.23
	CBPI—PIS (0–10)	3.8	2.6	0.01*	5.7	5.3	0.04*	<0.05*	3.9	2.8	0.01*	3.1	2.4	0.07	0.33	3.5	2.4	0.01*	4.0	3.0	0.63	0.22
	COI—Stiffness (0–16)	4.6	4.1	0.87	5.3	4.0	0.78	0.48	4.6	3.9	0.33	4.9	3.6	0.50	0.39	4.0	5.7	0.82	4.8	4.4	0.10	0.48
	COI—Function (0–16)	5.7	5.3	0.2	5.7	5.3	0.79	<0.05*	5.0	5.2	0.21	4.9	4.4	0.75	<0.01*	4.0	5.4	1.0	3.3	3.8	0.39	0.01*
	COI—Gait (0–20)	6.9	5.1	0.19	7.8	6.8	0.69	0.19	5.7	5.5	0.11	6.7	4.6	0.18	0.16	4.4	5.4	0.87	6.5	5.8	0.01*	0.46
	COI—QOL (0–12)	5.3	3.3	0.39	5.0	3.7	<0.01*	0.57	5.1	2.8	0.02*	4.3	2.7	0.49	0.59	4.7	2.6	0.09	4.4	3.2	0.03*	0.25
	COI—Overall score (0–64)	22.4	19.1	0.04*	22.9	19.7	0.75	0.26	20.1	15.7	0.29	20.3	14.5	0.53	0.14	15.7	14.9	0.1	20.9	18.9	0.09	0.21
	LOAD (0–52)	16.4	13.1	0.22	18.1	13.5	0.47	0.88	13.1	12.4	0.72	15.1	9.4	0.07	0.17	13.1	12.4	0.88	16.0	12.0	0.03*	0.07
Digital Thermography	DV (°. mean±SD)	25.3	1.5	0.36	24.4	0.8	0.36	1.0	26.1	1.2	0.04*	26.3	1.6	0.02*	0.68	25.6	1.4	0.89	25.1	0.9	0.57	1.0
	DV max (°. mean±SD)	25.2	2.1	0.88	25.9	0.7	0.31	1.0	27.4	1.4	0.14*	27.6	1.1	<0.01*	0.02*	26.9	1.4	0.74	26.5	0.9	0.78	1.0
	Lt (°. mean±SD)	29.8	2.2	<0.01*	29.5	2.3	<0.01*	<0.01*	28.4	1.8	<0.01*	29.0	2.6	<0.01*	<0.01*	27.3	1.8	0.21	28.7	2.3	<0.01*	<0.01*
	Lt max (°. mean±SD)	33.9	1.2	<0.01*	33.7	1.6	<0.01*	<0.01*	30.5	1.9	<0.01*	31.4	2.6	<0.01*	<0.01*	29.7	1.9	0.13	31.2	2.3	<0.01*	<0.01*
Synovial fluid	IL-1 (pg/mL. mean±SD)	122.9	108.9	0.05	186.6	104.5	0.24	1.0	159.6	59.1	0.13	169.1	55.3	0.36	1.0	184.2	68.5	0.25	145.1	33.5	0.07	1.0
	CRP (mg/mL. mean±SD)	0.48	0.9	0.18	0.1	0.2	0.43	1.0	0.4	0.8	0.36	0.0	0.0	0.42	1.0	0.0	0.0	0.5	0.0	0.0	0.18	1.0
Weight-bearing	Symmetry Index (mean±SD)	18.9	12.2	0.04*	13.3	8.6	<0.01*	<0.01*	27.4	12.1	0.29	14.0	13.5	0.02*	0.02	27.0	27.9	0.51	20.7	22.7	0.01*	0.22
	Deviation (mean±SD)	2.5	1.917	0.2	1.8	2.3	0.02*	<0.01*	2.72	2.27	0.29	2.3	2.1	0.04*	<0.01*	2.61	2.973	0.55	2.0	2.8	0.04*	0.75

CBPI—Canine Brief Pain Inventory; CRP—C-reactive protein; COI—Canine Orthopedic Index; DV—dorsoventral view; HVAS—Hudson Visual Analogue Scale; IL-1 –Interleukin 1; LOAD—Liverpool Osteoarthritis in Dogs; LT—lateral view; PIS—Pain Interference Score; PSS—Pain Severity Score; QOL—Quality of Life.

* indicates significance when comparing the value registered by a group at an evaluation day with T0 (p), and comparing both groups at each follow-up day (P).

**Table 3:** Time to return to baseline values for weight-bearing distributions (symmetry index and deviation) and CMIs, calculated with Kaplan-Meier estimators and compared with the Breslow test.

		Treatment			
Variable	Breslow test	CG		THG	
		mean±SD	95% CI	mean±SD	95% CI
Symmetry Index	0.003*	47.0±11.8	23.8±70.2	96.0±12.8	70.9±121.1
Deviation	0.022*	44.8±12.1	21.1±68.5	81.8±14.7	52.9±110.6
HVAS	0.269	48.7±12.4	25.4±73.9	66.1±14.2	38.3±93.9
PSS	0.065	63.2±17.2	29.6±96.8	90.2±17.6	55.7±124.7
PIS	0.000*	8.4±0.4	7.7±9.0	118.6±16.3	86.7±150.5
LOAD	0.000*	40.7±10.6	19.9±61.4	124.3±15.9	93.1±155.5
Stiffness	0.004*	64.7±16.9	31.4±97.9	130.8±11.6	108.1±153.5
Function	0.046*	65.4±13.4	39.2±91.6	112.6±15.6	81.9±143.2
Gait	0.001*	52.7±14.6	23.9±81.4	117.0±15.1	87.5±146.5
QOL	0.044*	60.9±15.0	31.4±90.4	119.3±17.5	85.0±153.6
COI	0.146	52.7±13.4	26.5±78.9	85.6±15.9	54.4±116.9

COI—Canine Orthopedic Index; HVAS—Hudson Visual Analogue Scale; LOAD—Liverpool Osteoarthritis in Dogs; PIS—Pain Interference Score; PSS—Pain Severity Score; QOL—Quality of Life.

* indicates significance.

### Radiographic findings

Frequency of different radiographic findings observed in CG and THG, at the initial evaluation, are presented in Table 4. In THG, an increase in the frequency of flattened or shallow acetabulum, with irregular outline was observed at 90 and 180 day (p<0.05). Increased new bone formation on the acetabulum and femoral head and neck was also observed at 90 day (p<0.05), as the frequency of CCO at 180 day (p<0.05).

**Table 4:** Frequency of radiographic findings in the Control (CG) and Treatment Groups (THG) in a ventrodorsal and frog-leg views, at the initial evaluation.

Radiographic finding	THG				CG			
	Present		Absent		Present		Absent	
Irregular wear on the femoral head, making it misshapen and with a loss of its rounded appearance	20	100%	0	0%	17	85%	3	15%
Flattened or shallow acetabulum, with irregular outline	16	80%	4	20%	11	55%	9	45%
Caudolateral curvilinear osteophyte (CCO)	8	40%	12	60%	5	25%	15	75%
New bone formation on the acetabulum and on femoral head and neck	17	85%	3	15%	20	100%	0	0%
The angle formed at the cranial effective acetabular rim is worn away	18	90%	2	10%	18	90%	2	10%
Subchondral bone sclerosis along the cranial acetabular edge	20	100%	0	0%	19	95%	1	5%
Circumferential femoral head osteophyte (CFHO)	8	40%	12	60%	3	15%	17	85%

In CG, an increase in the frequency of flattened or shallow acetabulum, with irregular outline, an increase was observed at 90 (p<0.01) and 180 day (p<0.01), compared with the initial evaluation day. In the THG, patients without CCO had higher LOAD (p<0.05), PSS (p = 0.02), PIS (p = 0.01), stiffness (p = 0.01), function (p<0.05), gait (p<0.01), QOL (p<0.01) and COI scores (p<0.01), and lower HVAS scores (p<0.01). At 15 day, they had lower mean and maximal thermographic evaluations on a DV (p = 0.01 for both) and mean on a Lt view (p = 0.04), and higher LOAD (p<0.01), stiffness (p = 0.04), function (p<0.01), gait (p<0.01), QOL (p<0.01) and COI scores (p<0.01). Again at the 30 and 90 day evaluations, those without CCO at the initial evaluation had higher HVAS (p<0.05 and p<0.01, respectively), and lower PSS (p<0.01 for both), PIS (p<0.01 and p = 0.02, respectively), LOAD (p<0.01 for both), stiffness (p = 0.04 and p = 0.02, respectively), function (p<0.01 and p = 0.04, respectively), gait (p<0.01 and p = 0.04, respectively), QOL (p<0.01 for both) and COI scores (p<0.01 and p = 0.02, respectively). At the final evaluation, they had only higher HVAS score (p = 0.03). In CG, animals without CCO at the initial evaluation did now show significant differences with those that did. However, at the 30-day evaluation, they had higher mean thermographic evaluation on a Lt view (p = 0.04), better joint flexion (p = 0.03), lower IL-1 and higher CRP concentration levels (p = 0.04 for both). On the 90 day evaluation, animals without CCO had lower maximal thermographic evaluation on a Lt view (p<0.05) and lower CRP values at 180 days (p = 0.02).

In the THG, patients without CFHO at the initial evaluation had lower pedometer counts (p<0.01) and lower HVAS (p = 0.04) and higher PIS scores (p = 0.03) on that day. At 8 day, they had higher body weight (p = 0.02) and higher deviation (p = 0.01) and symmetry index (p<0.05). At 15 day, they had higher body weight (p = 0.04), lower pedometer count (p<0.01) and higher LOAD score (p<0.05). Again at 30 day, these patients showed higher deviation (p = 0.04) and symmetry index (p<0.01). At 90 day, they had higher deviation (p = 0.03), symmetry index (p<0.01) and higher synovial IL-1 concentration (p = 0.01). At the final evaluation day, they had lower pedometer counts (p = 0.02). In CG, joints without CFHO at the initial evaluation had higher joint extension (p<0.01) and HVAS (p = 0.02), lower PSS (p = 0.01) and PIS scores (p = 0.03) at the 8 day evaluation. At 15 day, they had higher mean thermographic values on a Lt view (p = 0.02), lower PSS (p = 0.02) and PIS scores (p<0.05). This higher mean thermographic values on a Lt view was again observed at 30 day (p = 0.01) and higher HVAS scores (p = 0.02) at 90 day. At the final evaluation, they had higher maximal thermographic values on a Lt view (p = 0.04), and lower PSS (p = 0.05) and PIS scores (p<0.03).

### Comparisons by sex

In the THG, females had lower symmetry index (p<0.01) and lower synovial CRP concentration (p<0.01). At the 8 day evaluation day, females were lighter than males (p = 0.04) and had higher synovial IL-1 levels (p = 0.04). Additional differences were observed at 15 days, with female dogs having lower mean and maximal temperatures on a DV (p<0.01 for both) and Lt views (p = 0.04 and p<0.01, respectively). At 30 days, females had higher pedometer counts (p<0.01) and, at 90 days, lower mean and maximal temperatures on a DV view (p<0.05 and p<0.01, respectively). At the final evaluation day, no differences were observed between sexes. Female dogs of CG had significantly lower body weight throughout the study (p = 0.01). In the first evaluation, they also showed higher values in all thermographic evaluations (p<0.01) and lower PIS scores (p = 0.04). Again at 8 days, higher thermographic evaluations were recorded (p<0.01), except maximal value on a Lt view, as higher joint extension values (p<0.01). A higher joint extension was again observed in female dogs at 15 days (p = 0.04), with lower PIS scores (p = 0.03). At the 30 days evaluation females again showed higher thermographic maximal values on an LT view max (p<0.01). Female dogs at 90 days had lower thigh girth (p = 0.03) and lower PSS and PIS scores (p = 0.01). At the final evaluation day, they had higher extension values (p = 0.02), higher HVAS (p = 0.02), and lower PSS (p<0.01) e PIS (p<0.01), stiffness (p = 0.02), function (p = 0.02), gait (p<0.01), QOL (p = 0.02) and COI (p = 0.01) scores.

### Comparisons by bodyweight

Comparing animals in THG with a weight below the mean value of the sample, had higher deviation (p = 0.03), symmetry index (p = 0.04), mean and maximal values on thermography DV view (p<0.01 and p = 0.02, respectively), lower thigh girth (p<0.01) and lower synovial IL-1 levels (p = 0.02). At 8 days, they had lower thigh girth (p<0.01) and lower PSS (p = 0.01), PIS (p<0.01) and stiffness scores (p = 0.01). After 15 days, lighter patients had higher symmetry index (p = 0.03) and lower thigh girth (p<0.01). At 30 days, these patients had higher pedometer counts (p = 0.01) and lower thigh girth (p<0.01). At the 90-day evaluation, they had higher mean and maximal values on thermography Lt view (p = 0.02 and p<0.01, respectively), lower thigh girth (p<0.01) and higher joint flexion (p = 0.03). 30 At the final evaluation day, lighter animals had lower mean and maximal values on thermography a DV (p<0.01 for both) but higher on a Lt view (p<0.01 for both), lower thigh girth (p<0.01) and higher joint flexion (p = 0.03). In CG, lighter patients registered had lower PIS scores (p = 0.04) at the initial evaluation. These patients, at the 8 day evaluation, had higher thermographic mean and maximal values on a DV (p = 0.03 and p = 0.02, respectively), lower thigh girth (p = 0.01), and higher stiffness (p = 0.03), function (p<0.01), gait (p = 0.03) and COI scores (p<0.01). Significant differences were again observed at 15 days, with lighter patients showing lower thigh girth (p = 0.04) and HVAS (p<0.05), and higher stiffness, function, gait QOL e COI scores (p<0.01). They also had lower CRP concentrations at 30 days (p = 0.04) and higher HVAS scores (p = 0.02). At 90 days, they had lower thigh girth (p<0.01) and IL-1 levels (p = 0.02) at 90 days. At the final day of evaluation, lighter animals showed higher mean thermographic values on a DV view (p<0.01), and higher joint flexion (p = 0.02) and extension (p<0.01).

### Comparisons by age

Considering patients above or below the mean age in the THG, at the initial evaluation younger patients had higher mean and maximal temperature on a Lt view (p<0.01 for both), and lower PSS (p = 0.01), stiffness (p = 0.04), function (p = 0.02), gait (p = 0.02), QOL (p = 0.04), and COI scores (0.01). After treatment, at 8 days, they had lower deviation (p = 0.03) and symmetry index (p = 0.04), higher mean temperature on a Lt view (p<0.01), lower synovial IL-1 concentration (p = 0.04), and lower PIS (p<0.02) and function scores (p = 0.03). At 15 days, they had higher mean and maximal temperature on a DV view (p<0.01 for both) and Lt view (p<0.01 for both), and lower PIS (p<0.01), stiffness (p = 0.03), gait (p<0.01) and COI score (p = 0.04). After 30 days, younger animals had higher mean and maximal temperature on a Lt view (p = 0.01 for both), higher HVAS (p<0.01), and lower PSS (p<0.01), PIS (p<0.01), LOAD (p<0.01), stiffness (p<0.01) and QOL scores (p<0.01). Differences regarding CMI scores were observed again at 90 days, with the same patients having higher HVAS (p<0.01), and lower PSS (p = 0.01), PIS (p<0.01) and QOL scores (p = 0.01). At the final evaluation, patients bellow the mean age value had higher pedometer counts (p<0.01), lower deviation (p = 0.02) and SI (p<0.01), higher HVAS (p<0.01), and lower PSS (p<0.01), PIS (p<0.01), LOAD (p<0.01), stiffness (p<0.01), function (p = 0.04), gait (p<0.01), QOL (p<0.01) and COI scores (p<0.01). In the CG at the initial evaluation, younger patients had higher maximal values on the thermographic Lt view (p = 0.04), lower LOAD (p = 0.02), stiffness (p<0.01), function (p<0.01), gait (p<0.01) and COI (p<0.01) scores. After 8 days, they showed lower SI (p<0.01), higher maximal values on the thermographic Lt view (p = 0.02) and lower LOAD (p = 0.04), stiffness (p<0.01), function (p<0.01), gait (p<0.01), QOL (p<0.01) and COI (p<0.01) scores. Again at 15 days, younger patients presented lower LOAD (p<0.01), stiffness (p<0.01), function (p<0.01), gait (p<0.01), QOL (p<0.01) and COI (p<0.01) scores. At the 30 day evaluation, they again presented improved evaluations in several parameters, with lower mean and maximal values on the thermographic DV (p<0.01 and p = 0.02, respectively) Lt view (p = 0.02, for the mean value), higher joint flexion (p = 0.01) and lower LOAD (p<0.01), stiffness (p<0.01), function (p<0.01), gait (p<0.01), QOL (p<0.01) and COI (p<0.01) scores. Better CMI scores was again observed at 90 days, specifically lower LOAD (p = 0.04), stiffness (p<0.01), function (p<0.01), gait (p<0.01), QOL (p<0.01) and COI (p<0.01) scores. At the final evaluation, patients bellow the sample mean age had lower deviation and SI (p = 0.03 and p<0.01, respectively), and stiffness (p<0.01), function (p<0.01), gait (p<0.01), QOL (p<0.01) and COI (p<0.01) scores.

## Discussion

OA is a leading cause of disability around the world, impacting the physical and mental well-being of populations, posing a substantial toll on healthcare and financial resources [66]. To our knowledge, this is the first study to describe the effect of a single injection of triamcinolone hexacetonide on several clinical, imaging and laboratorial signs in a naturally occurring canine osteoarthritis model, with a long follow up period.

There are some reports evaluating the effect of IA TH in humans. A 2-year follow-up study showed that TH has long-term safety, with no deleterious effects being observed deriving from IA administration [67–69]. Also, patients treated had significant increases in ROM and improvements in pain [67]. These improvements are noticeable with the results of the Kaplan Maier test for symmetry index, with results in SG taking significantly longer to return to baseline values. It was also observable with different scores, as function or stiffness. Comparing TH to a saline injection, TH (40mg) had higher effectiveness than the placebo group in the four weeks in terms of pain in movement, pain scale, and ultra-sound measurement of synovial hypertrophy [70]. Treatment with 20mg or 40mg of TH produced equal relapse after six months in patients with chronic polyarthritis and when treating medium-sized joints. With that in mind, since no difference in outcome was found between the compared doses, the authors advised that lower dose should be preferred, reducing pharmaceutical costs and metabolic side effects [71, 72]. As a whole, these reports present the overall safety and effectiveness of IA TH in the management of OA, measured with multiple validated CMI and other clinical evaluations. With this animal model, a single IA TH administration was able to significantly reduce weight-bearing changes in affected joints up to the 90-day evaluation compared with a control group. Besides changes in different CMI scores, particularly pain scores calculated with the CBPI, were observed. This is of particular interest, since pain is a hallmark of OA, and its characterization produces valuable data that may translate to humans [21, 73, 74]. Individual CMI scores in THG improved for a majority of animals were observed with several of the considered questionnaires, in many cases up to the last evaluation. A significant difference was also observed with the Kaplan Meier test for the majority of the considered scores. In contrast, patients in the CG had worse scores (meaning lower HVAS scores and higher values in the remaining considered CMIs scores) throughout the follow-up period, particularly with as time progressed. Our results are in line with previously described effects for TH, but since in dogs OA progresses faster while maintaining the same stages [9], it is possible that in humans results may be observed for a more extended period. Additionally, since patients who composed this sample are active working dogs, their musculoskeletal structures are under increased stress and effort [75], leading to an earlier decline in initially observed improvements. It would also be of interest to have intermediary follow-ups between the 90 and 180-day evaluations, in order to further precise the duration of treatment efficacy. Some patients in the CG also showed some improvements, particularly at the 8 and 15-day evaluations. It may be due to the natural evolution of osteoarthritis, with the possibility for spontaneous improvements in some stages of the disease. An additional possibility is related to the removal of cytokine loaded synovial fluid at the time of the evaluation and the posterior administration of saline, which can lead to an effect similar to a joint lavage. In fact, placebo saline injections have shown an effect in functional improvements that can last up to a 6-month follow-up [76].

IL-1 is commonly pointed out as the most important proinflammatory cytokine responsible for the catabolic events in OA [1, 49, 50]. Corticosteroids are, of the medications available for the treatment of OA, the ones with most potent anti-inflammatory activity, specifically through the downregulation of the synthesis of inflammatory mediators such as IL-1β, TNF-α and COX-2 in the synovial fluid [64, 77–79]. In this study, significant differences between CG and THG were only observed at the 8-day evaluation and, even though IL-1 levels were lower in both groups compared to the initial evaluation, levels in CG were lower. This shows that TH can reduce IL-1 levels, which can be partly responsible for its ability to improve OA clinical signs, but the removal of synovial fluid and the administration of saline is also able to do so. Despite this effect, IL-1 levels cannot be the sole responsible for OA clinical signs, since, despite lower IL-1 levels of CG, this group still had worse clinical signs.

Radiographic evaluation is a staple of OA monitoring. Previous OA animal studies have demonstrated a decrease in disease progression or a protective role of corticosteroids injections, based on histological and biochemical findings [18, 80–84]. A recent systematic review of canine models of OA induction concludes that the reports regarding its IA use appear to be unanimously positive, with lower doses with sustained joint concentrations having a protective effect [85]. In CG, the natural progression of the disease was observed as expected, and radiographic signs progressed throughout the follow-up period. In the THG, radiographic signs also progressed, particularly in the more advanced follow-up days. Although radiographic signs progressed, they seemed to be less severe in THG. We only characterized radiographic sings as present or absent, so this eventual protective role of IA TH may not be entirely recorded. The evaluation of CCO and CFHO is of particular clinical interest, as they represent early radiographic signs that predict the development of the clinical [42, 43, 86, 87]. Our results support this finding since animals with CCO or CFHO in both views showed worse clinical signs at the initial evaluations. Also, animals with these radiographic findings showed a worse response to treatment, despite THG having a positive evolution compared with CG. Digital thermography can assess inflammatory pain in osteoarthritic patients [47, 48]. Our findings showed mixed results regarding the thermographic evaluation. While, in some evaluation days, animals with higher temperatures recorded in different thermography evaluations corresponded to those patients with worse clinical signs, in other days, it did not. This may indicate that other characteristics may play an important role, e.g. the amount of muscle masses surrounding the joint, in this case, represented by thigh girth, or variations in body weight.

Documented risk factors for OA include higher bodyweight and increasing age [2]. To evaluate the effect of these factors, we considered results with a cut-off for weight and compared younger to older patients. Considering bodyweight at different cut-off points, heavier patients in both groups generally showed worse clinical signs, particularly with different CMI scores. This effect of body weight may also be responsible for the fact that females also had better CMI scores than males since they were also lighter. Similarly, patients with age above the mean sample age showed worse evaluations during the follow-up period and worse response to treatment in THG. This may reflect that older patients may have a more degenerate joint with more advanced OA-induced changes, and therefore worse clinical signs, with a reduced ability to show improvements in response to therapy.

Side effects of IA procedures are mainly related to discomfort from the procedure itself, localized pain post-injection and flushing. IA corticosteroids can also cause synovitis, in a reactive reaction called a steroid flare, with a described prevalence of 2–6% [4, 78, 88, 89]. More rare reported side effects include crystal-induced synovitis, calcification and steroid arthropathy [90]. We observed increased lameness in four patients, which spontaneously resolved within 48 hours. No additional medication was administered to the animals during the follow-up period. When compared to other therapeutic options, IA TH may be a more cost-effective option due to its lower cost [91].

## Conclusions

To our knowledge, this is the first study to describe the effect of a single injection of triamcinolone hexacetonide in a naturally occurring canine model, with a long follow up period. THG recorded significant improvements in weight-bearing up to the 90-day follow-up. Improvements were also observed with the considered CMIs, particularly pain scores. Lower thermographic values were registered in THG up to the last evaluation day. Age, sex, and radiographic findings did significantly influenced response to treatment.
