# Correction: The intra-articular administration of triamcinolone hexacetonide in the treatment of osteoarthritis. Its effects in a naturally occurring canine osteoarthritis model

**DOI:** 10.1371/journal.pone.0248082

**Published:** 2021-02-26

**Authors:** João C. Alves, Ana Santos, Patrícia Jorge, Catarina Lavrador, L. Miguel Carreira

There are errors in the Funding statement. The correct Funding statement is as follows: This work was supported by Centro de Investigação Interdisciplinar em Sanidade Animal (CIISA), Faculdade de Medicina Veterinária, Universidade de Lisboa, through Grant UIDB/00276/2020, from the Portuguese Foundation for Science and Technology (FCT) to LMC.
