# Expression of Concern: The intra-articular administration of triamcinolone hexacetonide in the treatment of osteoarthritis. Its effects in a naturally occurring canine osteoarthritis model

**DOI:** 10.1371/journal.pone.0276113

**Published:** 2022-10-07

**Authors:** The PLOS ONE Editors

After publication of this article [1], concerns were raised about possible overlap or re-use of datasets and potential data discrepancies across a group of closely related publications [1–8]. The studies published previously in [2], [4], and [6] were not cited and discussed in [1]. In following up on these issues, the following was noted with regard to the datasets reported in these studies:

- The thermographic image in Fig 5 of article [1] appears similar to the thermographic images in Fig 1 (left panel) of article [4] and in Fig 1 (left panel) of article [5].
- Articles [1], [3] and [6] report data from clinical metrology instruments following treatment of osteoarthritis with triamcinolone hexacetonide (THG) compared to a saline-treated control group (CG), with or without other treatment groups. The articles report the same aggregate body weight and age data (bodyweight 26.7 ± 5.2 kg; age 6.5 ± 2.4 years) for animals included in these studies. Article [1] reports a sample size of 20 animals, while articles [3] and [6] report a sample size of 50 animals.
- Table 2 of article [1] (n = 20 joints per group) reports several identical aggregate values for clinical metrology instrument scores and weight-bearing data on the day of treatment (T0) when compared to the data in Table 1 of article [3] (n = 20 joints per group) for THG and CG groups; the values differ for control group scores for PIS, gait, QOL, COI overall, deviation control, and for both THG and CG groups for symmetry index.
- The CG and THG data in Table 3 of article [1] (n = 20 joints per group) appear to be the same as in Table 2 of article [3] (n = 20 joints per group) with the exception of the Breslow test.

The corresponding author stated that these articles are part of a larger project which was run with a specific population of police working dogs, a relatively homogenous sample in terms of breeds, size, and conformation, and that articles [1] and [3] are part of the same project where they chose to follow the same time points and outcome measures to be able to compare results between all of the produced manuscripts. For that reason, the tables presenting the procedures conducted and timepoints are similar. The corresponding author further stated that article [1] focuses on triamcinolone hexacetonide (TH) and presents multiple outcome measures not presented in articles [3] and [6]. It compares TH to a negative control on a translational approach, where group results are compared. Articles [3] and [6] compare multiple treatments, but the approach evaluated the impact of treatment, age, breed, sex, and OFA hip score on the observed outcomes. This approach focuses on individual results, whereas the Kaplan-Meier test is simply to show that difference occurs in order to build the Cox regression model.

The corresponding author also stated that the thermographic images in Fig 5 of article [1], Fig 1 of article [4] and Fig 1 of article [5] are images of different dogs which were specifically prepared to present the same dorsoventral view, where the same temperature scale was applied, and the same emissivity. The corresponding author commented that these are standardized images for similar patients, with the same disease and degree of disease, and are supposed to be very similar.

The corresponding author has indicated that because of legal restrictions, the authors are unable to provide the *PLOS ONE* Editors with the underlying individual level data and the original, unaltered, uncropped thermographic image files for this study. The published route for requesting access to the underlying data does not seem to be functional at the time of publication of this notice.

The corresponding author stated that the population of working dogs in article [7] does not overlap with the populations in articles [1–6] and [8]. Where differences are observed between the datasets, it has not been possible to confirm the accuracy of the data reported in this *PLOS ONE* article [1].

In light of the questions about the datasets not being fully resolved, the *PLOS ONE* Editors issue this Expression of Concern.
